# Two-step hierarchical binary classification of cancerous skin lesions using transfer learning and the random forest algorithm

**DOI:** 10.1186/s42492-024-00166-7

**Published:** 2024-06-17

**Authors:** Taofik Ahmed Suleiman, Daniel Tweneboah Anyimadu, Andrew Dwi Permana, Hsham Abdalgny Abdalwhab Ngim, Alessandra Scotto di Freca

**Affiliations:** Department of Electrical and Information Engineering, University of Cassino and Southern Lazio, Cassino, 03043 Italy

**Keywords:** Random forest, Machine learning, Deep learning, Class imbalance, Hierarchical classification, Cancerous skin lesions

## Abstract

Skin lesion classification plays a crucial role in the early detection and diagnosis of various skin conditions. Recent advances in computer-aided diagnostic techniques have been instrumental in timely intervention, thereby improving patient outcomes, particularly in rural communities lacking specialized expertise. Despite the widespread adoption of convolutional neural networks (CNNs) in skin disease detection, their effectiveness has been hindered by the limited size and data imbalance of publicly accessible skin lesion datasets. In this context, a two-step hierarchical binary classification approach is proposed utilizing hybrid machine and deep learning (DL) techniques. Experiments conducted on the International Skin Imaging Collaboration (ISIC 2017) dataset demonstrate the effectiveness of the hierarchical approach in handling large class imbalances. Specifically, employing DenseNet121 (DNET) as a feature extractor and random forest (RF) as a classifier yielded the most promising results, achieving a balanced multiclass accuracy (BMA) of 91.07% compared to the pure deep-learning model (end-to-end DNET) with a BMA of 88.66%. The RF ensemble exhibited significantly greater efficiency than other machine-learning classifiers in aiding DL to address the challenge of learning with limited data. Furthermore, the implemented predictive hybrid hierarchical model demonstrated enhanced performance while significantly reducing computational time, indicating its potential efficiency in real-world applications for the classification of skin lesions.

## Introduction

Skin cancer is a global health concern characterized by the uncontrolled proliferation of abnormal skin cells, often triggered by DNA damage from prolonged exposure to ultraviolet (UV) radiation [1–3]. According to the World Health Organization, environmental factors influencing UV exposure primarily include latitude and altitude, with higher UV levels closer to the equator and at higher altitudes where there is less atmosphere to absorb UV radiation [4]. Diagnosis of skin cancer typically involves a dermatologist’s clinical examination supported by dermoscopic imaging and confirmed by skin biopsy. However, global health disparities based on geographic location have resulted in a shortage of dermatologists and pathology laboratory facilities in rural areas [5, 6], hindering timely access to skin cancer detection and contributing to increased morbidity and mortality rates [7–9]. Skin cancer severity varies based on lesion type and stage. For instance, nodular melanoma can rapidly progress and metastasize if untreated, leading to complications such as bleeding, infection, and skin scarring, impacting quality of life [10–12]. Therefore, accurate diagnosis of skin lesions is crucial for timely and effective treatment [13–15]. However, diagnosing skin lesions presents challenges due to reliance on expert visual analysis of dermoscopy images, which suffers from interobserver variability and subjective interpretation [16, 17]. Additionally, the high resolution and heterogeneity of skin lesions, along with factors like hair causing clutter, further complicate dermoscopy diagnosis. Thus, there is a need for advanced computer-aided diagnosis (CAD) techniques, potentially coupled with Internet of Medical Things (IoMT) devices, to automate screening and early detection of skin cancer [18, 19]. Big data, computer vision, and artificial intelligence (AI) technologies, including machine and deep learning (DL) techniques, have been employed in various medical contexts, including disease diagnosis and treatment optimization [20]. In dermatology, these techniques are utilized for skin lesion classification, melanoma detection, and diagnosing skin diseases. However, achieving precise classification of melanoma skin lesions from images is essential for CAD systems to facilitate effective diagnosis.

Previous methodologies relied on handcrafted features extracted from images to capture essential visual characteristics, along with conventional classifiers [21, 22]. More recent approaches utilize deep convolutional networks for hierarchical feature learning from images. Deep neural networks have been employed alongside conventional classifiers [23, 24] and in end-to-end systems [25–27]. Despite significant research advancements, further improvements in diagnostic accuracy (ACC) have been hindered by several limitations [28]. Main challenges include the inadequate sample size of publicly accessible datasets, their unbalanced nature, and the requisite pre-processing operations for classifying various skin lesions, such as enhancement and segmentation. Training a deep neural network entails learning from a dataset with several million parameters based on its structure. The network’s parameter count directly influences the dataset size required. In cases of limited samples, DL networks pre-trained on larger datasets like ImageNet and transfer learning methods are viable options [25, 29]. Additionally, small sample sizes and image artifacts may predispose the model to overfitting. To address this, common techniques include employing dropout and applying data augmentation. However, the question posed in this paper pertains to the correlation between overfitting and parameter count. In such instances, opting for more traditional classifiers like support vector machines (SVMs) [30], k-nearest neighbors (k-NNs) [31], random forest (RF) [32], and logistic regression (LR) [32] becomes viable, as they require fewer parameters.

Skin images present a significant challenge due to the heterogeneity of skin lesions, characterized by varying sizes and positions within the images, along with the presence of clutter, further complicating the diagnostic process using dermoscopy. Pre-processing techniques can enhance the classification of diverse skin lesions. Hosny et al. [23] introduced an approach based on convolutional neural networks (CNNs) for skin lesion classification, beginning with a pre-processing step where the region of interest (ROI) is segmented. Their study demonstrated that accurately identifying the ROI through integrated pre-processing and segmentation significantly improves classification results compared to existing state-of-the-art methods. Methods employing segmentation to identify ROIs from color skin images can be supervised, contingent upon the availability of ground truth annotations [33]. However, annotating skin lesions necessitates dermatological expertise, which may not always be readily available. Manual annotation of skin lesions is time-consuming, requiring meticulous review by dermatologists to identify and classify lesions accurately. Nevertheless, achieving precise segmentation without labeled data poses a formidable challenge [34]. Unsupervised segmentation struggles to delineate object boundaries, particularly for skin lesions exhibiting high variability in shape, size, and appearance, while being sensitive to imaging artifacts and lighting variations. Moreover, interpreting segmentation results is subjective, with multiple plausible segmentations possible for a given image. Additionally, segmentation algorithms can be computationally intensive, impractical for real-time applications or large-scale datasets.

Regarding the proposed system’s intended purpose, integrating such models into mobile devices proves impractical, particularly in remote and rural areas with limited computational resources. Furthermore, the feasibility of cloud-based intelligent diagnosis is constrained to developed countries or regions with advanced infrastructure. Given the urgent need for portable, cost-effective, and automated diagnoses with minimal computational requirements, our research emphasizes a single-model-based approach.

The issue also pertains to the severely imbalanced distribution of sample numbers among different skin lesion classes. In this scenario, one class significantly outperforms the others, leading to its dominance. Consequently, a model trained on such data may exhibit bias toward predicting the majority class more frequently, raising concerns about false negatives (FNs).

FNs are particularly concerning in medical diagnosis, as failing to detect melanoma can result in serious negative outcomes. Various techniques can address class imbalance to mitigate false-negative issues. These include adjusting class weights, utilizing different evaluation metrics such as precision (PRC), recall (REC), and F1 score, or employing specific algorithms designed for imbalanced data [35]. For instance, Yao et al. [36] proposed a multi-weighted loss method to overcome class imbalance by adjusting weights during conventional training of deep layers. Alsahafi et al. [27] developed a bootstrapping technique for dataset balancing. This method involves regular sampling with replacement and weighting of samples based on the number of images in each class.

Nevertheless, optimizing the performance of deep neural networks remains crucial for accurately classifying skin lesions, regardless of dataset limitations. Both recent techniques necessitate a training procedure that involves significant changes to network weights. However, considering the constraints of insufficient dataset sample size discussed earlier, the use of pre-trained models was deemed preferable.

Considering these factors, a two-step hierarchical binary classification approach is proposed to address challenges associated with class imbalance issues. This approach allows for a more focused treatment of distinct critical issues, particularly evident in the International Skin Imaging Collaboration (ISIC 2017) dataset, where the focus shifts from more numerous classes to less numerous ones. Furthermore, both upsampling and downsampling techniques were employed at each step to rectify the imbalance in class samples. The evaluated CNN models as feature extractors include VGG16 (VGG) [37], ResNet50 (RNET) [15], and DenseNet121 (DNET) [38]. These networks were chosen to assess the impact of different structures and parameter counts. Additionally, a novel CNN architecture with a reduced number of parameters was introduced. The recognition times and performances of the four models during the predictive phases were compared. Two experiments were conducted: one utilizing the CNNs in an end-to-end system and the other detaching the last layers of the CNN and employing the aforementioned traditional methods as classifiers. The study demonstrated the effectiveness of the two-step hierarchical model, particularly when DNET was combined with the RF classifier.

The remainder of the paper is organized as follows: Related works subsection provides a brief overview of the research activities related to our study. Methods section outlines the dataset used and details the proposed binary two-step architecture. Results section presents the experimental results, and Discussion section compares and discusses the achieved results. Finally, Conclusions section presents the concluding remarks.

### Related works

Multiple studies have focused on classifying skin lesions. For instance, Esteva et al. [39] demonstrated the direct classification of skin lesions from images using a single CNN-trained end-to-end method, utilizing pixels and disease labels as inputs. The model was trained on open-access dermatology repositories, including the ISIC Archive, Edinburgh Dermofit Library, and Stanford Hospital dataset. It achieved ACC of 72.1%, tested across both tasks with 21 certified experts. This demonstrated the capability of AI to classify skin cancer at a level comparable to dermatologists. However, a notable limitation of this study is its relatively low ACC, suggesting that exploring pre-trained models may enhance the performance of the model. Furthermore, Mahbod et al. [40] suggested three pretrained deep models, namely AlexNet, VGG16, and ResNet-18, as deep feature generators. Subsequently, the collected features were utilized to train multiclass nonlinear SVM classifiers. Multiple classifiers were trained for each network, and the class scores were averaged to obtain the final classification results. LR was also employed to transfer the SVM scores to probabilities for evaluating the classification outcomes. The image dataset used for training, validation, and testing was the ISIC 2016 competition, with the training set of the ISIC 2017 competition utilized for training the classifiers. The proposed method achieved commendable classification performance, yielding an area under the receiver operating characteristic (ROC) curve of 83.83% or melanoma classification and 97.55% for seborrheic keratosis classification [40]. Increasing the number of pre-trained networks could potentially enhance these results, and training the model on original or large-resolution images might be preferable for resizing the images to prevent the loss of useful information. In another study [41], the authors devised a densely connected convolutional network technique known as ARDT-DenseNet for skin lesion classification. Each ARDT block comprised dense blocks, transition blocks, attention, and residual modules. The size of the parameters of the densely connected network suggested in this study decreased by half compared to a residual network with the same number of convolutional layers, while maintaining the ACC of skin lesion classification. The ARDT-DenseNet model was tested using ISIC 2016 and ISIC 2017 datasets. In skin lesion classification with ISIC 2016, the proposed technique achieved ACC of 85.7% and an area under the curve (AUC) of 83.7%, whereas with the ISIC 2017 dataset, an ACC of 87.8% and an AUC of 95.7% were attained [41]. The model’s performance demonstrated significant improvements despite the reduced number of parameters compared to similar models, and these results could potentially be further enhanced by leveraging pre-trained models.

Ramamurthy et al. [42] proposed a two-stage network for skin disease detection utilizing atrous residual convolutional networks. This approach involves segmentation and classification models for skin lesion detection. Classification was conducted on seven different classes of skin lesions from the HAM10000 dataset, yielding an ACC and PRC of 89.27% and 89.06%, respectively [42]. While the method demonstrates balanced interclass performance and precise segmentation, the complexity of the model may result in longer training times and increased computational resource requirements.

Karthik et al. [43] developed Eff2Net, utilizing EfficientNetV2 with the efficient channel attention (ECA) block. The ECA block replaced the standard squeeze and excitation blocks in the EfficientNetV2 architecture, leading to a significant decrease in trainable parameters without compromising performance. This method was employed to classify four types of skin diseases: acne, actinic keratosis, melanoma, and psoriasis. Despite utilizing fewer parameters, the model achieved a lower overall testing ACC of 84.70%. Thurnhofer-Hemsi et al. [44] introduced an ensemble of enhanced CNN for skin lesion classification, incorporating a regularly spaced test-time shifting method. This technique involves using shifted versions of the test image, which are then fed into each classifier within an ensemble. The final result is a combination of the classifier outputs. Results from the HAM10000 dataset surpassed those of simple DL networks without shifting, achieving ACC and F-scores of 83.5% and 68.8%, respectively. While this method utilizes fewer parameters, the ensemble approach increases computational complexity.

Aswathanarayana and Kanipakapatnam [45] proposed a saliency-based level set with an enhanced boundary indicator function for effective segmentation of skin cancer. This method exhibits effectiveness in detecting skin cancer boundaries even under low illumination and intensity conditions. Following segmentation, features from these images were extracted using GoogLeNet, which utilizes sparse connections for optimal feature extraction. Classification was then conducted using a multi-class SVM on the ISIC-2017 dataset, achieving an ACC of 98.74%. While this method yielded promising results, its sensitivity to image segmentation quality could impact overall ACC. Hosny et al. [25] employed transfer learning with a modified AlexNet to classify seven classes of skin lesions using the ISIC 2018 dataset, achieving an ACC of 98.70%. In a subsequent study [23], they proposed a DCNN-based method integrating preprocessing, segmentation, and augmentation, utilizing architectures such as AlexNet, ResNet101, and GoogleNet. This approach showcased an enhanced classification process, particularly with the modified GoogleNet, achieving an ACC of 98.14% on the ISIC 2017 dataset. However, its reliance on high-quality image preprocessing and segmentation limits practical applicability in less-controlled environments.

Another study [27] utilized sliding dot product filters instead of sliding filters along the horizontal axis to classify skin lesions. This approach employed a residual deep CNN and multiple convolution filters for multi-layer feature extraction and cross-channel correlations. Converting the dataset from images and labels to vectors of images and weights helped address class imbalance. Testing on the ISIC-2019 and ISIC-2020 datasets demonstrated an ACC and sensitivity of 94.65% and 70.78%, respectively, for the ISIC-2019 datasets and 99.05% and 96.57%, respectively, for the ISIC-2020 datasets.

## Methods

This section provides an overview of the materials, sources of the skin lesion image dataset, and methodologies utilized to accomplish the two-step hierarchical binary classification. As outlined in the introduction, this study aims to demonstrate the efficacy of the two-step hierarchical architecture in addressing common challenges encountered in publicly available skin lesion datasets, namely small size and data imbalance. Initially, the hierarchical binary architecture’s effectiveness in mitigating imbalance issues is elucidated, emphasizing the prioritization of numerous classes before focusing on fewer classes. Next, the data preparation process is detailed to specify the input data for the two subsequent models. Finally, the deep and machine learning (ML) models utilized in each hierarchical step are outlined. To tackle the challenge of a small dataset size, the utilization of pretrained models in end-to-end systems was chosen, along with their use as feature extractors in conjunction with traditional ML classifiers. The selection of these models was substantiated by evidence from published sources [15, 46–50].

### Image dataset

**Fig. 1:**
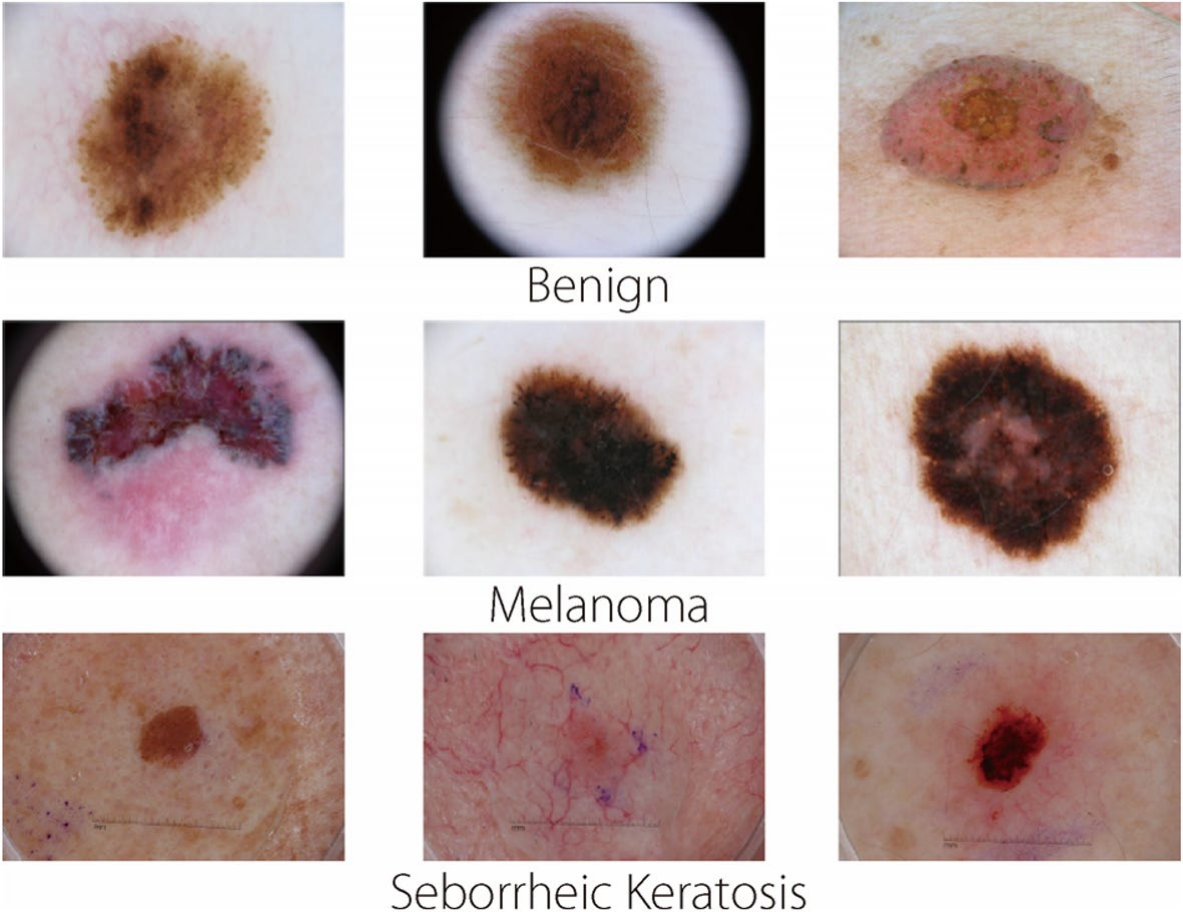
Examples of different types of skin lesion

The image database utilized in this project comprised 2000 lesion images in JPEG format sourced from the ISIC 2017 dataset challenge. The database encompassed images of three distinct lesion types: melanoma (374 images), seborrheic keratosis (254 images), and benign lesions (1372 images). Illustrations of different lesion types are depicted in Fig. 1. To ensure precise labeling and evaluation, the dataset also provided corresponding ground-truth labels for all images. Each image was assigned a label corresponding to its lesion type based on the image ID, facilitating supervised learning and performance assessment of the classification models.

### Proposed architecture

**Fig. 2:**
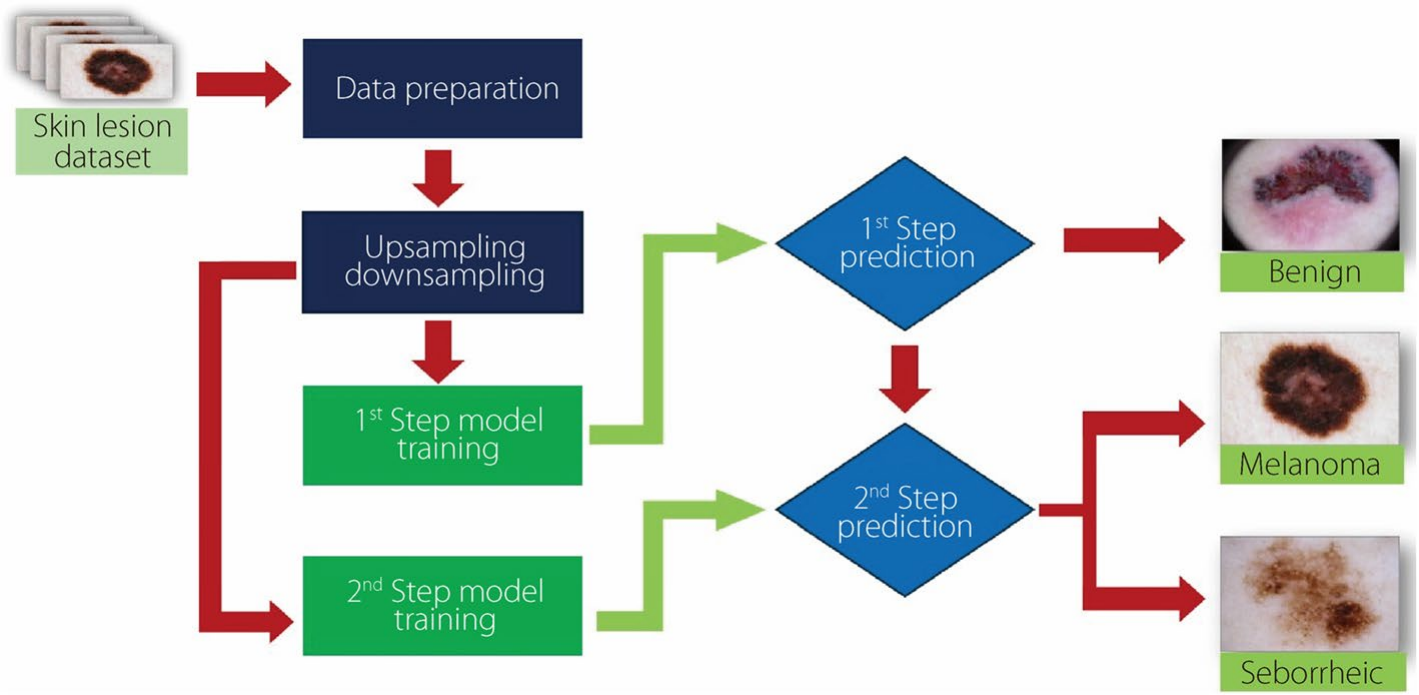
Schematic of the proposed two-step classification process

The proposed architecture employs a two-step hierarchical binary classification approach to improve classification performance and tackle challenges stemming from class imbalance. Figure 2 illustrates the framework of the two-step classification process. This process unfolds sequentially, with the first step focusing on classifying the majority class (benign), followed by the classification of the remaining classes (melanoma *vs* seborrheic keratosis) in the second step. In the initial classification step, the emphasis lies on distinguishing between the benign class and the combination of the melanoma and seborrheic keratosis classes (benign *vs* others). This step aids in identifying instances most likely to be benign. Subsequently, in the second step, samples predicted as ‘others’ (non-benign) in the first step undergo further classification to ascertain whether they belong to the melanoma or seborrheic keratosis class. This two-step approach enhances the ACC of classification results by performing binary classification in two sequential steps. It is essential to note that although these steps are described sequentially, they are executed simultaneously, and the final classification results are presented without an intermediate ‘Others’ classification.

The proposed two-step hierarchical binary classification approach is delineated using mathematical formulations guiding each step. In the first classification step, denoted as *Y*_1,_ the output distinguishes between ‘Benign’ and ‘Others.’ Given that the primary objective is to identify melanoma, the class containing melanoma is labeled as the positive one; thus, in this initial step, it corresponds to the ‘Other’ class.

Mathematically, this can be expressed as:

1 $${Y}_{1}={f}_{1}\left(X\right)$$

where *X* represents the input dataset containing skin lesion images. The goal of this step is to identify instances likely to be categorized as either ‘Benign’ or ‘Others.’

In the second classification step, when *Y*_1_ is ‘Other’, i.e.,* Y*_1_ = 1, the classification is conducted to ascertain whether the sample belongs to the ‘Melanoma’ or “Seborrheic Keratosis” class. Mathematically, this step can be represented as:

2 $$Y_2=f_2\left(X\right)\;only\;when\;Y_1=1$$

where *Y*_2_ denotes the second binary classification label. To enhance model performance and mitigate the influence of misclassified data during training, the second phase selectively utilizes only correctly classified ‘Other’ data containing seborrheic keratosis and melanoma from *Y*_1_. Consequently, the final classification label *Y* is either *B* (benign), *S* (seborrheic keratosis), or *M* (melanoma), based on the following combinations of *Y*_1_ and *Y*_2_:

3 $$Y=\left\{\begin{array}{c}B\;when\;Y_1=0\\S\;when\;Y_1=1,Y_2=0\\M\;when\;Y_1=1,Y_2=1\end{array}\right.$$

This hierarchical approach enables focused and sequential classification, effectively addressing challenges associated with class imbalances and intrinsic variability in skin lesions. Consequently, the overall effectiveness of the approach relies on the reduction of false positives (FPs) in both steps.

### Data preparation

Considering the significant class imbalance in the dataset, both upsampling and downsampling techniques were employed to address this issue. Initially, in the first classification step, the Melanoma and Seborrheic Keratosis classes were merged and labeled as ‘Others.’ This amalgamated class, originally comprising 628 samples (374 melanoma and 254 seborrheic keratosis), was then upsampled to 1000 samples using random sampling techniques. Concurrently, the benign class was downsampled to 1000 samples using random sampling. This process was facilitated through the utilization of the ‘resample’ function from the sklearn utility library, implementing a single step of the bootstrapping procedure. Consequently, this sampling methodology yielded a total of 2000 samples, with the ‘Benign’ and ‘Others’ classes having an equal number of samples, thus ensuring a balanced class distribution for the second step of classification.

### DL modules

The effectiveness of the proposed two-step hierarchical architecture was demonstrated through the utilization of ML and DL modules. To address the challenge posed by a small dataset, a variety of deep networks with differing structures and parameter numbers were employed: VGG, RNET, and DNET. Specifically, three established models known for their efficacy in image classification tasks, including those involving skin lesions [51, 52], were selected, alongside the development of a custom CNN architecture. Table 1 summarizes the main differences among these models.

**Table 1:** Deep neural network used

DNN	Layer number	Parameter number	Residual block
VGG	16	138	No
RNET	50	23	Yes
DNET	121	7	Yes
CNN	5	0.5	No

**Fig. 3:**
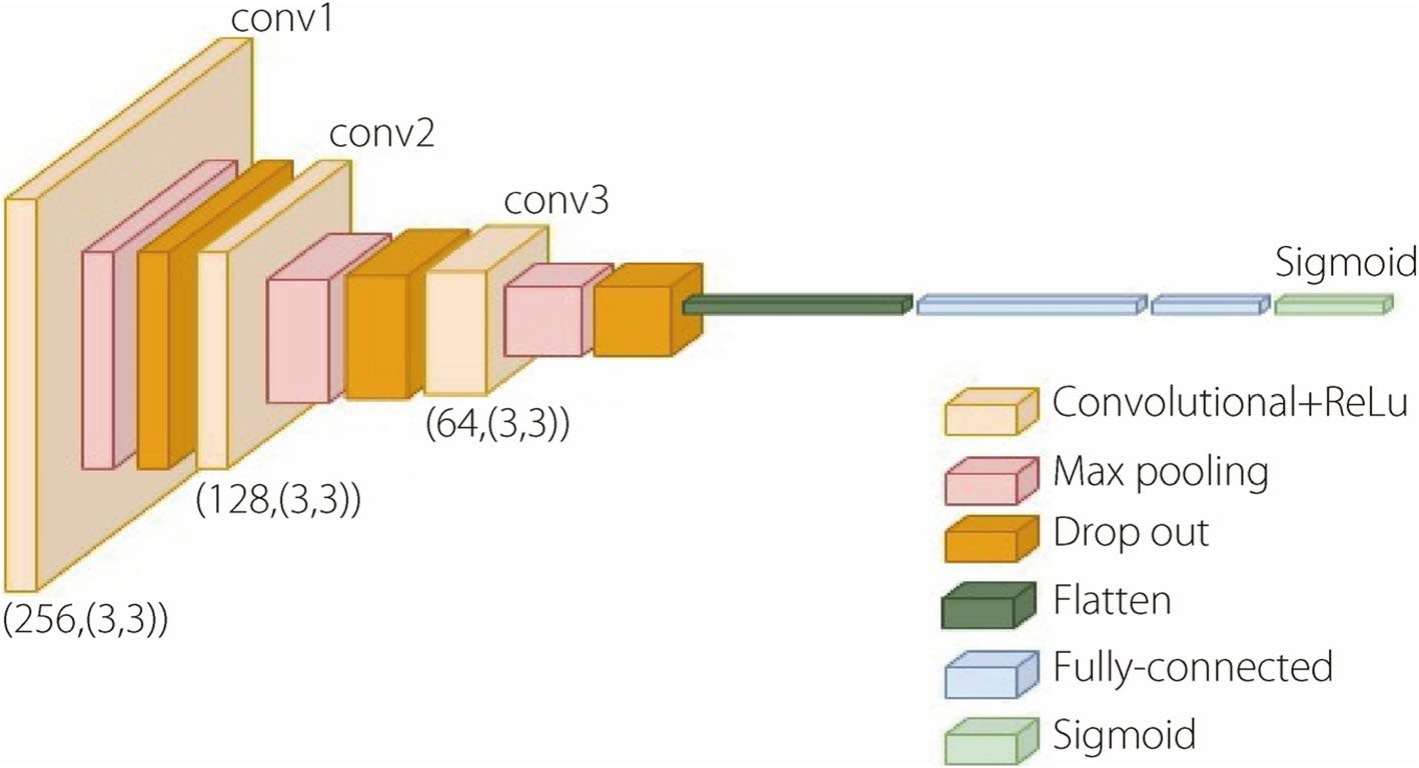
Visual representation of the network’s architecture

VGG’s architecture served as the baseline for the initial exploration of the skin lesion classification task. In contrast, RNET’s utilization of skip connection layers within the residual learning framework addressed challenges such as the vanishing gradient problem, rendering it proficient at capturing intricate features within heterogeneous skin lesion images. DNET’s dense connectivity, feed-forward approach, and efficient parameter sharing further enhanced its capability to identify patterns, offering particular advantages for skin lesion classification. All established models were initialized with pre-trained weights from ImageNet. To preserve the pre-trained features during fine-tuning, all layers of the pre-trained models were set as non-trainable. Additionally, a flattened layer was introduced to convert the output of the pre-trained models into 1D vectors, which were subsequently fed into densely connected layers to augment the capacity for learning task-specific features. The final dense layer incorporated a sigmoid activation function for binary classification. Binary cross-entropy loss and the Adam optimizer were employed for model compilation, both of which are well-suited for binary classification tasks. Leveraging pre-training, a moderately high number of layers could be selected for the three networks. A custom CNN was devised to evaluate the hierarchical system, even employing a non-pretrained network. This custom architecture was designed following the guidelines generated by the training phase of our data on the AutoKeras generator [53]. The CNN architecture comprised sequential layers constructed using basic blocks of convolutional layers. Configured with a specified number of filters (256, 128, and 64) and a filter/kernel size of 3 × 3, the custom CNN architecture is depicted in Fig. 3.

The configuration was determined through an automated technique, AutoKeras, which searches for the optimal DL architecture. This technique relies on only two input parameters: the maximum number of trials and the number of epochs. In this study, the maximum number of trials is set to 16 with 15 epochs, resulting in 16 potential architectures. The model demonstrating the best performance in terms of ACC was selected for further use. To introduce non-linearity and enhance the model’s ability to learn complex patterns, the activation function ‘ReLu’ was employed. Max-pooling layers were integrated into the network architecture to reduce the spatial dimensions of the feature maps, allowing the model to focus on the most critical features while simultaneously reducing the number of parameters. By downsampling the feature maps, these layers enhance the model’s robustness and efficiency. To address overfitting, dropout layers were incorporated into the network. Dropout regularization was utilized to prevent the model from overly relying on specific features by randomly deactivating a certain percentage of neurons during training. This approach promotes better generalization and reduces the risk of overfitting. The final output of the model was obtained by flattening the 2D feature maps into a 1D vector, which was then fed into a fully connected layer for the final classification. The first fully connected layer consisted of 32 units, corresponding to half the size of our image. The ‘ReLu’ activation function was applied, and the number of class units in the final layer was set to 1, indicating the binary classification task. The ‘sigmoid’ activation function was chosen for the final layer, as it is suitable for binary classification, providing output probabilities for the two classes. It is noteworthy that the same network architecture was employed in training both steps of the hierarchical classification, albeit trained separately for each step. This approach ensures consistency and comparability between the two stages of the classification process, thereby enhancing the overall classification performance.

### ML module

In the machine-learning module, SVM, k-NN, LR, and RF were selected due to their versatility in handling diverse data types and their robust performance in classification tasks, including skin lesions, as documented in the literature [15, 46–50]. SVM’s adaptability to both linear and nonlinear tasks, along with its effectiveness in high-dimensional spaces, aligns well with the complex nature of skin lesion classification. k-NN, relying on instance-based learning and proximity to neighbors, excels in capturing local patterns. LR’s simplicity and interpretability make it suitable for binary classification tasks. RF’s ensemble learning approach, combining decision trees and proficiency in handling nonlinear relationships, contributes to robust predictions in skin lesion classification.

To extract meaningful features from the data, the same CNN and pre-trained models employed in the DL modules were utilized. For each pre-trained model and the proposed CNN, the output of the last layer was flattened and used as a feature extractor, which was then inputted into these classifiers. Additionally, feature engineering techniques were applied to enhance model performance. During the feature extraction and transformation stage, pre-trained models and convolutional filters were employed to extract features from the input data, effectively capturing spatial patterns and hierarchical representations within the images. These learned features provide diverse information regarding skin lesion characteristics. To ensure a fair comparison and prevent features with larger magnitudes from dominating the classification process, the standardized scaler method was applied to each extracted feature from the various models. This involved subtracting the mean and dividing it by the standard deviation, thereby accounting for variations and bringing the features to a similar scale. Furthermore, principal component analysis (PCA) was employed to address the challenge of high-dimensional feature spaces and mitigate potential overfitting in ML modules.

## Results

Two distinct experiments were conducted to validate the effectiveness of the proposed architecture. Initially, the aforementioned DL models were employed in an end-to-end architecture for both the first and second steps. In the second experiment, the DL models acted as feature extractors, while the previously mentioned ML methods served as classifiers. Thus, in the latter case, each DL method was paired with one of the four ML classifiers, resulting in 16 combinations. Each combination of DL and ML was then applied in both the initial and subsequent steps of the two-step hierarchical architecture to demonstrate its effectiveness.

For both experiments, the dataset was split using the class-wise splitting method, dividing the dataset so that the first 70% of images from each class were assigned to the training set, while the remaining 30% were allocated to the testing set, maintaining class-wise proportions. This fixed split ensured consistency across the different groups during model training. Furthermore, to prevent potential bias in the data order, the dataset was shuffled after splitting. The training set was utilized to train the 2-step hierarchical binary classifiers using fivefold validation at each step. Conversely, the test set was used to evaluate the entire two-step predictive model constructed using the best previously learned classifier. Thus, creating a predictive model for real-life applications is facilitated. In each experiment, the classifier rules used in the first and second stages were identical. During the testing phase, the model that achieved the best performance among the five previously learned models was assessed.

The performance of each model was evaluated using fivefold cross-validation. During the neural network training process, a batch size of 32 was employed, indicating that the model was updated based on 32 images simultaneously. This batch-based training approach optimizes memory usage and computational efficiency. Training was conducted for 100 epochs, implying that the entire dataset was processed 100 times during the training phase for each model.

Various performance metrics were utilized, including ACC, PRC, REC, F1-score, AUC, and balanced ACC (classification). These metrics are essential for evaluating model effectiveness, with PRC ensuring the ACC of positive predictions, REC emphasizing the model’s ability to capture positive instances, and F1-score providing a balanced assessment. Additionally, a comparison between the best models from the two experiments was performed using balanced multiclass accuracy (BMA), a critical measure for evaluating classification model performance. This metric represents the average ACC across all classes, considering the imbalanced nature of the dataset. Higher BMA values indicate better overall classification performance of the project. Finally, to ascertain the applicability of this predictive model to real-life scenarios, the overall prediction times were compared.

### First experiment

**Fig. 4:**
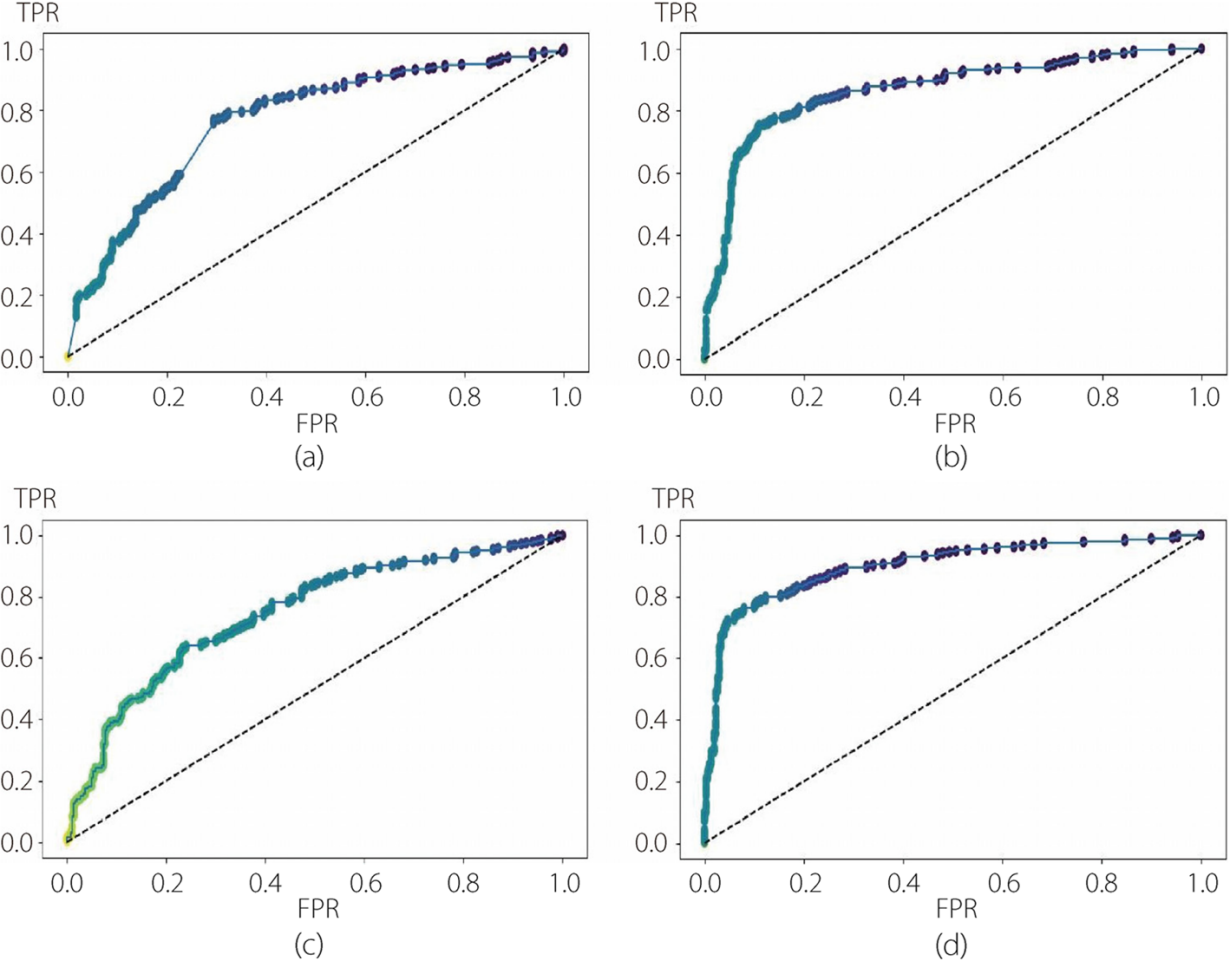
ROC curves for each deep model used in the first step of the first experiment. **a** CNN; **b** VGG; **c** RNET; **d** DNET

**Fig. 5:**
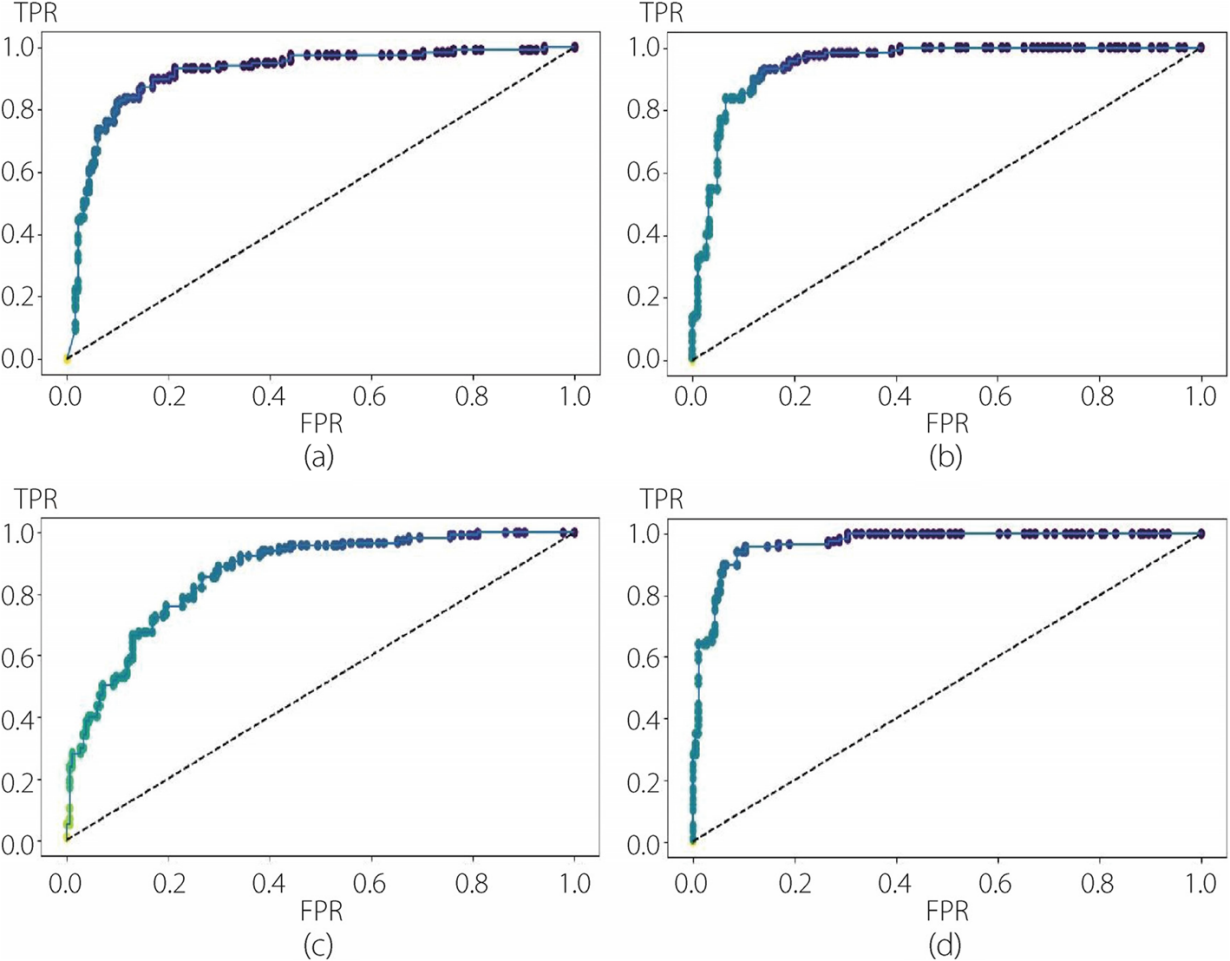
ROC curves for each deep model used in the second step of the first experiment. **a** CNN; **b** VGG; **c** RNET; **d** DNET

The performance evaluation of the DL modules involved training the CNN and three pre-trained models using fivefold cross-validation. Table 2 presents the cross-validation results of the models for each step, with the average and standard deviation values of the five replicates shown in the last two rows of the table. Subsequently, the test set was evaluated using the aforementioned metrics, and the results obtained for each step are presented in Table 3. The tables indicate that DNET outperformed the other models, with VGG achieving the second-best average ACC. To complement the tabular results, Figs. 4 and 5 illustrate the ROC curves of the first and second steps, respectively. The ROC curves reveal a greater learning challenge in the first step compared to the second. Additionally, they underscore the superiority of DNET over the other models, which is consistent with the findings in the tables.

**Table 2:** Cross-validation results from the first experiment

First step					Second step			
	CNN	VGG	RNET	DNET	CNN	VGG	RNET	DNET
Onefold	0.6793	0.8657	0.7457	0.8323	0.7989	0.8941	0.8086	0.9430
Twofold	0.7029	0.8307	0.6993	0.8320	0.8011	0.9487	0.7414	0.9071
Threefold	0.6779	0.8571	0.6965	0.8493	0.8500	0.9071	0.8143	0.9071
Fourfold	0.6685	0.8057	0.7621	0.8578	0.8643	0.8929	0.7714	0.9286
Fivefold	0.6821	0.7836	0.7318	0.8500	0.7338	0.9353	0.7842	0.9424
Average	0.6821	0.8286	0.7271	**0.8443**	0.8096	0.9156	0.7840	**0.9256**
SD	0.0113	0.0307	0.0257	**0.0103**	0.0459	0.0225	0.0264	**0.0160**

**Table 3:** Performance evaluation of the first experiment on the test set

First step					Second step			
	CNN	VGG	RNET	DNET	CNN	VGG	RNET	DNET
ACC	0.7000	0.7967	0.6800	**0.8250**	0.8704	0.8837	0.7807	**0.9136**
PRC	0.6546	0.7730	0.6617	**0.8197**	0.8362	0.8060	0.7576	**0.8421**
REC	0.8467	0.8400	0.7367	**0.8333**	0.8291	0.9231	0.6410	**0.9572**
FS1	0.7383	0.8051	0.6972	**0.8264**	0.8326	0.8606	0.6944	**0.8960**
AUC	0.7000	0.7967	0.6800	**0.8250**	0.8629	0.8909	0.7553	**0.9216**
BA	0.7000	0.7967	0.6800	**0.8250**	0.8629	0.8909	0.7553	**0.9216**

*BA* Balanced accuracy

In addition, Table 4 presents the values of the confusion matrix for each DL model in both the first and second steps. Each value in the table was calculated by dividing by the total number of samples in the test set. As previously noted, the effectiveness of the entire system in the medical field hinges on reducing FPs. FNs can have severe consequences, particularly in the case of melanoma detection, leading to negative and potentially serious outcomes.

**Table 4:** Classification results from the first experiment in terms of TP, TN, FP, and FN divided by the total number of samples in the test set

	First step				Second step			
	**TP**	**TN**	**FP**	**FN**	**TP**	**TN**	**FP**	**FN**
CNN	0.4233	0.2767	0.2233	0.0767	0.3223	0.5481	0.0631	0.0664
VGG	0.4200	0.3767	0.1233	0.0800	0.3588	0.5250	0.0864	0.0300
RNET	0.3683	0.3117	0.1883	0.1317	0.2492	0.5317	0.0797	0.1395
DNET	0.4167	0.4083	0.0917	0.0833	0.3721	0.5415	0.0698	0.0166

*TP* True positive, *TN* True negative

Considering this, Table 4 demonstrates the most significant reduction in FPs in the first step with VGG, while DNET achieved the second-best reduction, trailing the top performer by only 0.33%. The superior performance of DNET in the first step can be attributed to its optimal reduction in FPs. In the second step, DNET exhibited the best reduction, surpassing the second-best reduction achieved by VGG by 1.34%.

Consequently, based on the results in Table 4, which highlight the best reduction of FNs, it can be inferred that the top-performing model is RNET.

Despite conceding in the initial step, the two-step hierarchical model utilizing DNET as the base model achieved an overall improvement in false-negative reduction of 1.01 compared to the second-best reduction achieved by VGG.

### Second experiment

**Fig. 6:**
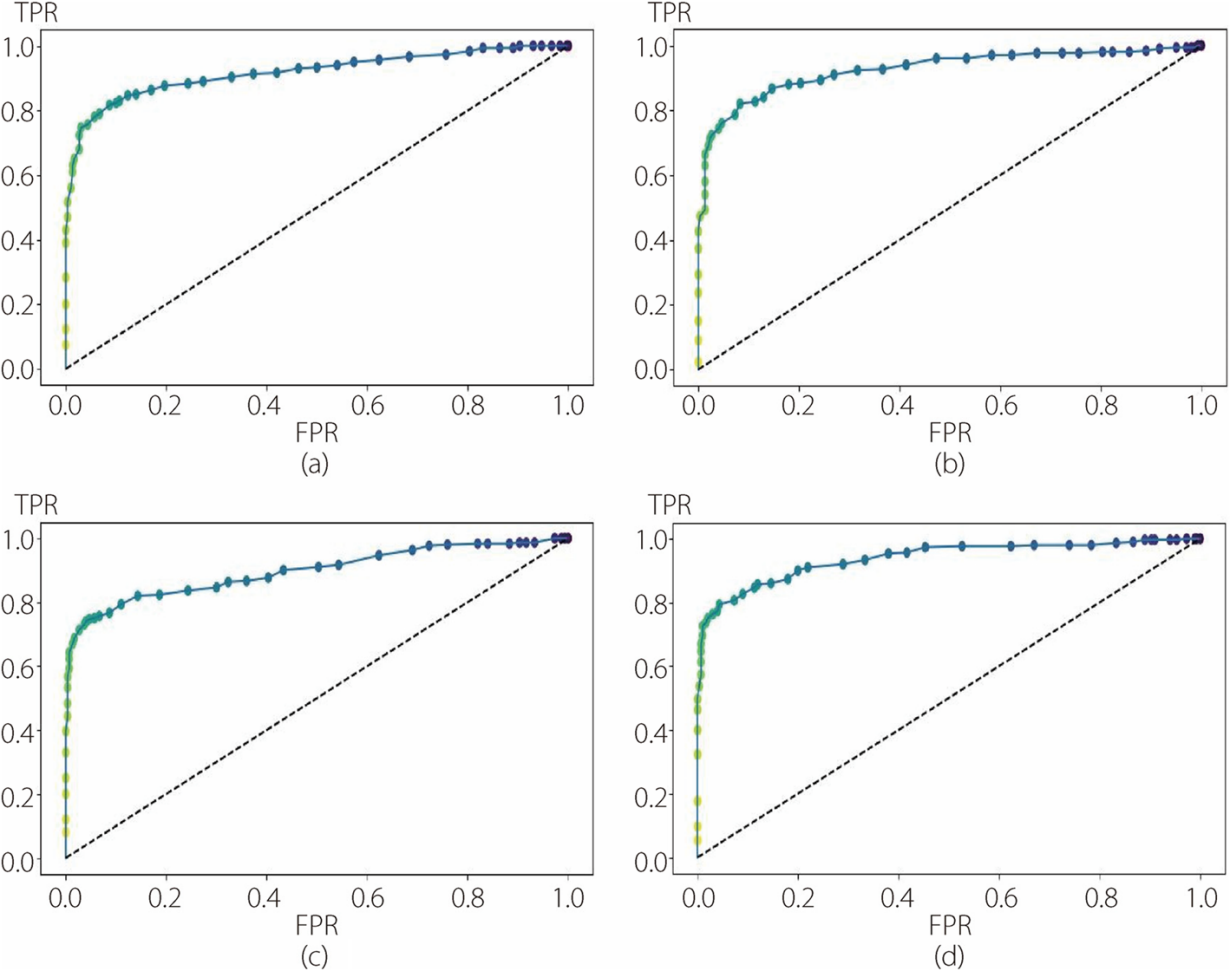
ROC curves for the RF with different feature extractors used in the first step of the second experiment. **a** CNN; **b** VGG; **c** RNET; **d** DNET

**Fig. 7:**
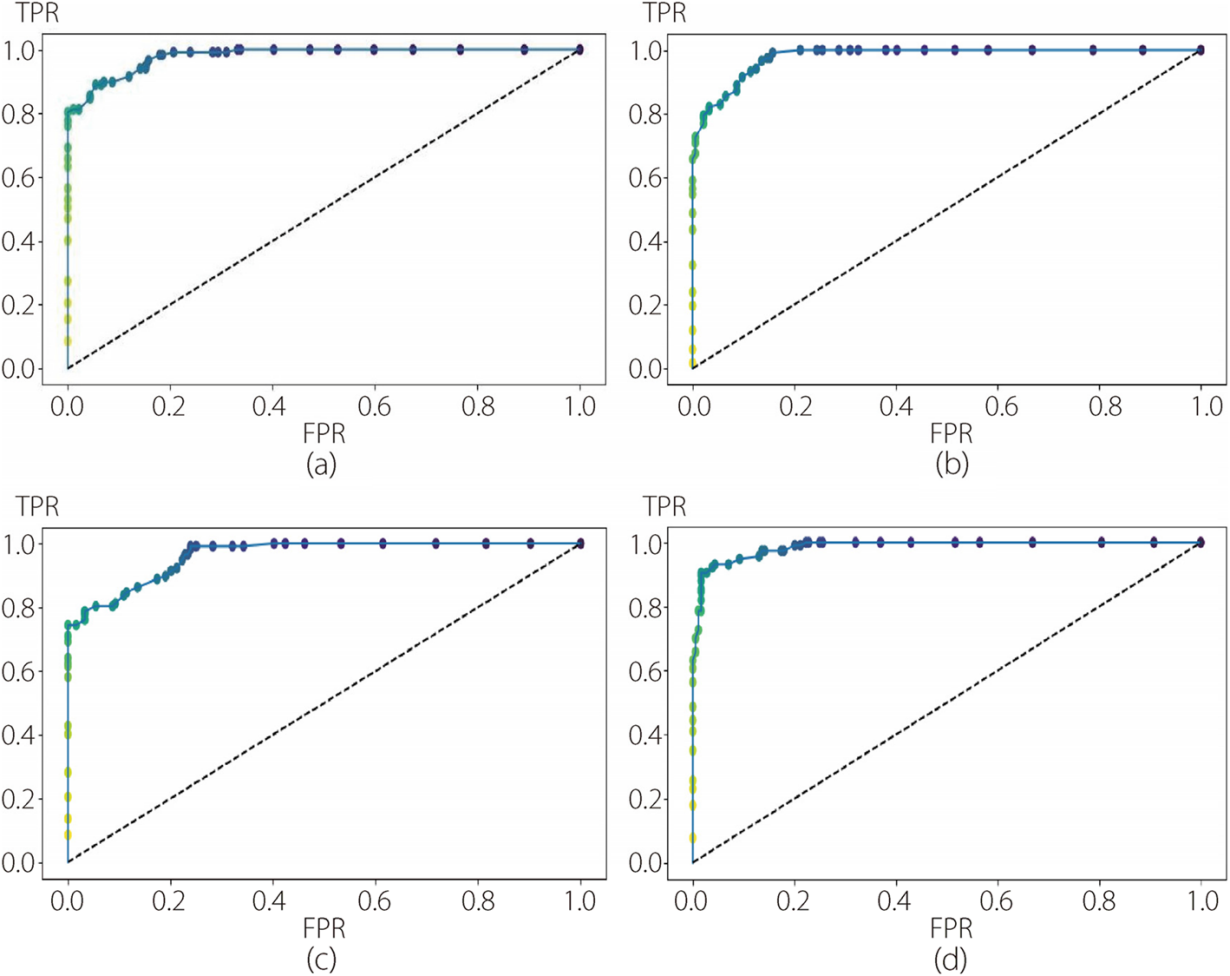
ROC curves for the RF with different feature extractors used in the second step of the second experiment. **a** CNN; **b** VGG; **c** RNET; **d** DNET

To evaluate the performance of the ML methods, fivefold cross-validation was employed for each step of the 2-step hierarchical binary classification. This method enabled the assessment of the effectiveness of the four different classifiers (RF, SVM, k-NN, and LR) with each deep feature extractor (CNN, VGG, RNET, and DNET) in accurately classifying skin lesions. Consequently, in the second experiment, 16 possible model configurations were compared for each step. Before integrating the DL module with the ML classifier, PCA was utilized to tackle the challenge of high-dimensional feature spaces and alleviate potential overfitting in these ML modules. PCA is commonly applied in dimensionality reduction to capture the most salient patterns and variances in data by projecting them onto a lower-dimensional subspace. Careful consideration was given to selecting the appropriate number of components required for PCA, balancing the tradeoff between preserving information and reducing dimensionality. In this approach, the number of components was set to 50 for the first step of binary classification (benign *vs* others) and 70 for the second step (melanoma *vs* seborrheic keratosis). These values were determined to be optimal for the classification task after conducting several experiments and meticulous evaluations. Tables 5 and 6 present the cross-validation results for the first and second classification steps in the ML modules, respectively. The average and standard deviation values of the five folds are displayed in the last two rows of each subtable. It is evident that the RF classifier with DNET as the feature extractor exhibits the highest average cross validation of 83.00% and 90.56% for the first and second steps, respectively. Furthermore, it can be observed that RF is the best-performing classifier in both steps, irrespective of the choice of feature extractor. In Table 6, the SVM result closely approximates the RF result for the DNET feature extractor. Secondly, model evaluation of the test set was conducted using the aforementioned metrics, and the results obtained for each step are presented in Tables 7 and 8. Once again, the RF with DNET as the feature extractor demonstrated its effectiveness with accuracies of 85.67% and 94.68% in the first and second steps, respectively. Similarly, other metrics indicated the superior performance of the RF in accurately classifying skin lesions. This superiority exceeded 10% in all comparisons. Hence, RF reaffirms its capability to classify benign lesions accurately in the first step and distinguish non-benign lesions into melanoma or seborrheic keratosis in the second step. Based on this evidence, the RF classifier was chosen, and a more focused comparative analysis was conducted, specifically concerning the deep feature extractor. Figures 6 and 7 show the ROC curves of the first and second steps of the RF with different feature extractors.

**Table 5:** Cross-validation results from the first step of the second experiment

	CNN				VGG				RNET				DNET			
	RF	SVM	k-NN	LR	RF	SVM	k-NN	LR	RF	SVM	k-NN	LR	RF	SVM	k-NN	LR
Onefold	0.8143	0.6607	0.7143	0.6464	0.8250	0.6464	0.7321	0.6250	0.8107	0.6857	0.6571	0.6964	0.8357	0.7071	0.7536	0.7179
Twofold	0.7964	0.6643	0.6714	0.6571	0.8107	0.6536	0.6786	0.6821	0.7893	0.6821	0.6893	0.6857	0.8036	0.6821	0.7107	0.6893
Threefold	0.8000	0.6750	0.6893	0.6714	0.8536	0.6893	0.7464	0.6964	0.8250	0.6750	0.7321	0.6643	0.8500	0.7036	0.7250	0.6964
Fourfold	0.8143	0.6786	0.6571	0.6821	0.7857	0.6250	0.7000	0.6036	0.8036	0.6893	0.6929	0.6607	0.8107	0.6464	0.6786	0.6607
Fivefold	0.8071	0.7214	0.6893	0.7214	0.7929	0.6679	0.6429	0.6714	0.7571	0.6214	0.6821	0.6321	0.8500	0.7393	0.7500	0.7429
Average	0.8064	0.6800	0.6843	0.6757	0.8136	0.6564	0.7000	0.6557	0.7971	0.6707	0.6907	0.6679	**0.8300**	0.6957	0.7236	0.7014
SD	0.0073	0.0217	0.0019	0.0007	0.0243	0.0215	0.0372	0.0354	0.0231	0.0251	0.0024	0.0222	0.0195	0.0307	0.0275	0.0276

**Table 6:** Cross-validation results from the second step of the second experiment

	CNN				VGG				RNET				DNET			
	RF	SVM	k-NN	LR	RF	SVM	k-NN	LR	RF	SVM	k-NN	LR	RF	SVM	k-NN	LR
Onefold	0.8928	0.8357	0.8071	0.7714	0.8286	0.8214	0.7929	0.8071	0.8429	0.7429	0.7643	0.7286	0.8786	0.9071	0.8286	0.8643
Twofold	0.8929	0.7857	0.6929	0.7571	0.9357	0.8286	0.7286	0.8071	0.8929	0.7643	0.7929	0.7500	0.9143	0.8786	0.8000	0.8500
Threefold	0.8786	0.8357	0.7357	0.8214	0.8857	0.8571	0.7929	0.8143	0.8643	0.7643	0.8286	0.7857	0.8857	0.9071	0.8571	0.8643
Fourfold	0.9143	0.8143	0.7571	0.7857	0.9143	0.8214	0.8071	0.7786	0.9071	0.7857	0.7714	0.8214	0.9143	0.9000	0.8357	0.9286
Fivefold	0.8777	0.7482	0.7050	0.7914	0.8777	0.8058	0.7842	0.7770	0.8921	0.7770	0.8273	0.7842	0.9353	0.9209	0.7985	0.8633
Average	0.8913	0.8039	0.7396	0.7854	0.8884	0.8269	0.7811	0.7968	0.8798	0.7668	0.7969	0.7740	**0.9056**	0.9027	0.8240	0.8741
SD	0.0133	0.0334	0.0407	0.0216	0.0364	0.0169	0.0273	0.0158	0.0015	0.0145	0.0270	0.0320	0.0208	0.0138	0.0223	0.0278

**Table 7:** Performance evaluation of the first step of the second experiment on the test set

	CNN				VGG				RNET				DNET			
	RF	SVM	k-NN	LR	RF	SVM	k-NN	LR	RF	SVM	k-NN	LR	RF	SVM	k-NN	LR
ACC	**0.8487**	0.6867	0.7133	0.6850	**0.8500**	0.6617	0.6800	0.6517	**0.8183**	0.6600	0.6633	0.6517	**0.8567**	0.7300	0.7083	0.7217
PRC	**0.8355**	0.6912	0.7152	0.6867	**0.8513**	0.6620	0.6836	0.6522	**0.8184**	0.6610	0.6641	0.6596	**0.8567**	0.7307	0.7089	0.7218
REC	**0.8633**	0.6867	0.7133	0.6850	**0.8500**	0.6617	0.6800	0.6517	**0.8183**	0.6600	0.6633	0.6517	**0.8567**	0.7300	0.7083	0.7217
FS1	**0.8492**	0.6848	0.7127	0.6843	**0.8499**	0.6615	0.6784	0.6514	**0.8183**	0.6595	0.6630	0.6514	**0.8567**	0.7298	0.7082	0.7216
AUC	**0.8467**	0.6867	0.7133	0.6850	**0.8500**	0.6617	0.6800	0.6517	**0.8183**	0.6600	0.6633	0.6517	**0.8567**	0.7300	0.7083	0.7317
BA	**0.8550**	0.6867	0.7133	0.6850	**0.8500**	0.6617	0.6800	0.6517	**0.8183**	0.6600	0.6633	0.6517	**0.8567**	0.7300	0.7083	0.7217

**Table 8:** Performance evaluation of the second step of the second experiment on the test set

	CNN				VGG				RNET				DNET			
	RF	SVM	k-NN	LR	RF	SVM	k-NN	LR	RF	SVM	k-NN	LR	RF	SVM	k-NN	LR
ACC	**0.9236**	0.8538	0.7774	0.8173	**0.9037**	0.8439	0.8439	0.8007	**0.8704**	0.7741	0.8007	0.7874	**0.9468**	0.8738	0.8738	0.8239
PRC	**0.9123**	0.8534	0.7804	0.8162	**0.9034**	0.8447	0.8436	0.8001	**0.8698**	0.7724	0.7989	0.7345	**0.9471**	0.8755	0.8738	0.8237
REC	**0.8889**	0.8538	0.7774	0.8173	**0.9037**	0.8439	0.8439	0.8007	**0.8704**	0.7741	0.8007	0.7874	**0.9468**	0.8738	0.8738	0.8239
FS1	**0.9004**	0.8536	0.7785	0.8165	**0.9032**	0.8442	0.8437	0.8003	**0.8696**	0.7729	0.7984	0.7867	**0.9466**	0.8743	0.8738	0.8238
AUC	**0.9173**	0.8447	0.7713	0.8039	**0.8947**	0.8380	0.8349	0.7887	**0.8582**	0.7576	0.7809	0.7732	**0.9394**	0.8718	0.8672	0.8140
BA	**0.9062**	0.8538	0.7774	0.8173	**0.9037**	0.8439	0.8439	0.8007	**0.8704**	0.7741	0.7874	0.7874	**0.9468**	0.8738	0.8738	0.8239

Upon comparing Fig. 4 with Fig. 6, a substantial improvement in the first classification step achieved by the RF can be observed. This enhancement was reflected in the increased AUC for each pure DL method employed in the first experiment. This is also evident from the comparisons shown in Figs. 5 and 7, where each ROC curve from the second experiment demonstrates an improvement compared to the best ROC of the pure DNET in the first experiment. Moreover, for each combination analyzed, the best AUC value was obtained using RF as a classifier and DNET as a feature extractor. The specifics of FNs in Table 9 show that in the first step, the most substantial reduction is accomplished by VGG, with a margin of 1% compared to DNET. The superior performance of DNET at this stage is justified by its greater reduction in FPs, with a margin of 1.67% compared to VGG and 1.17% compared to CNN. In the second step, the RF with DNET as a feature extractor achieved the most significant reduction, saving 0.34% of the samples compared with the second-best reduction attained by VGG. Thus, in the second experiment, the best reduction in FNs was obtained using RNET.

**Table 9:** Classification results from the second experiment in terms of TP, TN, FP, and FN divided by the total number of samples in the test set

	First step				Second step			
	**TP**	**TN**	**FP**	**FN**	**TP**	**TN**	**FP**	**FN**
CNN + RF	0.4317	0.4150	0.0850	0.0683	0.3454	0.5781	0.0333	0.0434
VGG + RF	0.4400	0.4100	0.0900	0.0600	0.3323	0.5715	0.0200	0.0399
RNET + RF	0.4117	0.4067	0.0933	0.0883	0.3124	0.5581	0.0532	0.0763
DNET + RF	0.4300	0.4267	0.0733	0.0700	0.3522	0.5946	0.0165	0.0365

## Discussion

In this section, the results of the two experiments are compared with each other and with state-of-the-art methods using the ISIC 2017 dataset. Additionally, a computational time study was conducted to demonstrate the effectiveness of this system in real-time applications.

As shown in the previous tables and figures, the best-performing model in the first experiment achieved accuracies of 82.50% and 92.16% in the first and second steps, respectively. The RF classifier with DNET as the feature extractor achieved the best performance in the second experiment, with accuracies of 85.67% and 94.68% in the first and second steps, respectively. Thus, the model learned in the second experiment outperformed the first one, demonstrating a combination with margins of 3.17% and 2.52% for the first and second steps, respectively. A comparison between the two experiments demonstrated that the selection of pre-trained models and RF classifiers effectively addressed the challenge posed by a small dataset size.

Furthermore, RF has emerged as the most effective mitigation technique among ML classifiers. Unlike other ML methods, RF is an ensemble composed of multiple decision trees. In this case, the number of estimators is set to 50 in each hierarchical classification step. This value was chosen through experimentation and fine-tuning to strike a suitable balance between the model complexity and performance. The RF classifier performed well in handling high-dimensional feature spaces, particularly in scenarios with limited training data. In contrast, DL models excel at learning intricate features and patterns, particularly in image recognition tasks. They can automatically extract relevant features from the data, eliminating the need for manual feature engineering, which was adopted in this study. However, DL requires a large amount of training data to be generalized effectively and can be susceptible to overfitting when the training data are limited. In our case, the limited performance of the DL models was attributed to the relatively small dataset size, which did not provide sufficient training examples for the models to learn and generalize the complex features associated with different skin lesion types effectively. Among the DL models, DNET emerged as the best-performing model, likely due to its dense connectivity, where each layer receives input from all preceding layers. This dense connectivity enhances feature reuse and promotes gradient flow during training. Moreover, dense connectivity reduces the number of parameters compared to traditional architectures by reusing features. This can lead to more parameter-efficient models and alleviate issues such as vanishing gradients during training. Examining the values presented in Table 10, it is evident that DNET has the lowest parameter count compared to the other pre-trained models.

**Table 10:** BMA of both experiments

	First experiment	Second experiment
CNN	0.7597	0.8882
VGG	0.8450	0.8698
RNET	0.7113	0.8433
DNET	0.8866	**0.9107**
Average	0.8007	0.8780
SD	0.0689	0.0247

BMA was selected to evaluate the overall performance of the two-step hierarchical architecture. BMA is a crucial metric for evaluating the performance of a multi-class classification model. It represents the average ACC across all classes, considering the imbalanced nature of the dataset. Higher BMA values indicate a better overall classification performance of the project. To calculate the BMA, the ACC of each class was considered before averaging. In the first step of our 2-step hierarchical binary classification, the ACC for the ‘Benign’ class was calculated, whereas in the second step, the accuracies for the ‘Melanoma’ and ‘Seborrheic’ classes were calculated. The BMA was then obtained by averaging the accuracies across all classes.

Table 10 displays the optimal outcome achieved by the RF classifier using DNET as the feature extractor, attaining a BMA of 91.07%. This result underscores the effectiveness of the RF + DNET model in accurately classifying diverse skin lesions within this two-step hierarchical architecture. Furthermore, Table 10 indicates that the models from the second set of experiments exhibit a higher average BMA than those from the first set of experiments. This finding further validates the incorporation of traditional classifiers in conjunction with pre-trained models, effectively addressing the challenge posed by a small dataset size.

The performance of the proposed method was compared with that of existing methods utilizing the ISIC 2017 dataset. Table 11 presents a quantitative comparison with the methods discussed in related work that used the ISIC 2017 dataset. The proposed method surpasses all other approaches that do not involve segmentation, including [54], which performs segmentation. This validates the effectiveness of the proposed approach in addressing the significant challenges posed by imbalanced and small datasets. In the proposed architecture, segmentation is not performed to keep the model free from annotation-related issues. Moreover, because it avoids segmentation, this method is more suitable for real-time applications.

**Table 11:** Comparison between the results of the proposed and existing methods on the ISIC 2017 dataset

References	Classification	Segmentation	ACC (%)
Esteva et al. [39]	Single CNN	No	72.10
Wu et al. [41]	ARDT-DenseNet	No	87.80
Hosny et al. [55]	Alex-net	No	87.31
Pham et al. [56]	SVM	No	87.38
Mahbod et al. [24]	ResNet18 + SVM	No	87.30
Al-Masni et al. [54]	Different DCNN	Yes	81.57
Hosny et al. [23]	DCNN	Yes	98.14
Aswathanarayana and Kanipakapatnam [45]	MSVM	Yes	98.74
Our method	DNET + RF	No	91.07

To assess the applicability of this hierarchical architecture in real-time applications, a final comparison was conducted between the two experiments based on the prediction time. Table 12 lists the prediction times of the first and second sets of experiments, comparing the different DL models. It is noteworthy that in terms of prediction times, CNN consistently outperformed the other DL methods, with a margin of 80 ms compared to DNET in the first experiment and 21 ms in the second experiment. This can be attributed to the simplicity and shallower structure of the CNN compared to other neural networks. Furthermore, DNET achieved the second-best prediction time, highlighting the efficiency of its parameters.

**Table 12:** Predictive time of both experiments

	First experiment	Second experiment
CNN	32 ms/step	44 ms/step
VGG	128 ms/step	82 ms/step
RNET	142 ms/step	73 ms/step
DNET	112 ms/step	61 ms/step
Average	104 ms/step	65 ms/step
SD	49 ms/step	16 ms/step

Additionally, Table 12 indicates that the models from the second set of experiments exhibited shorter prediction times than those from the first set of experiments. This reaffirms that the utilization of RF classifiers with deep-learning models effectively reduces the prediction time of the entire architecture, thereby enabling its practical implementation in real-life applications.

## Conclusions

This study proposed a two-step hierarchical binary architecture to tackle the challenges inherent in the skin lesions dataset, namely class imbalance and small size. The effectiveness of the proposed architecture was demonstrated in mitigating class imbalance by addressing each step of a specific binary unbalanced problem. Additionally, an analysis of deep neural networks and traditional machine classifiers guided the selection of the best base model for our system. The utilization of a RF classifier and a pre-trained DNET as a feature extractor simplifies the complexity of the entire architecture, resulting in superior recognition performance and lower prediction time compared to other analyzed methods. This reduced complexity facilitates the deployment of the system in real-time applications and IoMT devices, thereby addressing the lack of timely access to skin cancer detection in rural communities.

Moreover, the proposed method outperforms existing methods that do not involve segmentation, including [54], which incorporates segmentation. The avoidance of complexity segmentation was deliberate to maintain control over the overall model. While the study yielded commendable results, notably proposing a fast model for making predictions and addressing the issue of an imbalanced dataset, it also acknowledged the limitations and challenges encountered during the experimentation process. Specifically, the reliance solely on the ISIC 2017 dataset and a focus on supervised learning contributed to dataset biases, highlighting the necessity for extensively annotated datasets for skin lesion classification. To mitigate these issues, future work will explore incremental hierarchical architectures.

## Data Availability

The data that support the findings of this study is the publicly available ISIC 2017 training dataset.

## Abbreviations

CNN: Convolutional neural network
CAD: Computer-aided diagnosis
IoMT: Internet of Medical Things
ISIC: International Skin Imaging Collaboration
UV: Ultraviolet
SVM: Support vector machine
k-NN: K-nearest neighbor
RF: Random forest
LR: Logistic regression
DNET: DenseNet121
RNET: ResNet50
VGG: VGG16
TP: True positive
FP: False positive
TN: True negative
FN: False negative
ROI: Region of interest
PCA: Principal component analysis
AUC: Area under the curve
ROC: Receiver operating characteristic
ECA: Efficient channel attention
ACC: Accuracy
PRC: Precision
REC: Recall
BA: Balanced accuracy
CV: Cross validation
BMA: Balanced multiclass accuracy
AI: Artificial intelligence
ML: Machine learning
DL: Deep learning
